# Waste Reduction Strategies: Factors Affecting Talent Wastage and the Efficacy of Talent Selection in Sport

**DOI:** 10.3389/fpsyg.2019.02925

**Published:** 2020-01-10

**Authors:** Kathryn Johnston, Joseph Baker

**Affiliations:** School of Kinesiology and Health Science, York University, Toronto, ON, Canada

**Keywords:** talent, talent selection, talent wastage, selection bias, cognitive bias, decision-making

## Abstract

Coaches are faced with the difficult task of identifying and selecting athletes to their team. Despite its widespread practice in sport, there is still much to learn about improving the identification and selection process. Evidence to date suggests selection decisions (at different competitive levels) can be inaccurate, bias driven, and sometimes even illogical. These mistakes are believed to contribute to “talent wastage,” the effect of a coach’s wrongful selection and/or deselection of an athlete to/from a team. Errors of this scale can lead to negative repercussions for all stakeholders involved and therefore deserve further exploration. It is the purpose of this paper to shed light on the potential factors influencing talent wastage and to illuminate possible psychological pitfalls when making decisions under uncertainty.

## Introduction

In an effort to predict and select the next athletic superstar, substantial resources (e.g., time, money, and energy) are invested with the hope of gaining an edge over the competition. Although there is some evidence to show improvements in the identification and selection of athletes (Tetlock, 2016), research suggests that accuracy rates for predicting athlete potential remain quite low (Abbott and Collins, 2004; Vaeyens et al., 2008; Koz et al., 2012; Schorer et al., 2017; Johnston et al., 2018).

The talent selection process typically takes place early in an athlete’s life and involves administrative personnel (such as a coach, scout, or talent identifier^1^), who are tasked with identifying and predicting future athletic success. Often a series of tests (primarily focused on the physical or physiological attributes of an athlete) combined with coach observations (Christensen, 2009; Schorer et al., 2017) are used to assess performance (Lidor et al., 2009). Following this assessment period, talent selectors make decisions regarding which athletes should be included (selected) or excluded (de-selected) from the team. To date, there does not appear to be a “gold standard” approach to talent selection; rather, there appears to be a high degree of variability in the techniques, protocols, and processes used for assessment and selection. Current approaches range from subjective preferences and intuition (Williams and Reilly, 2000; Christensen, 2009; Lund and Söderström, 2017) and the use of standardized testing batteries (e.g., 40 meter sprint, vertical jump, Gabbett, 2009; Wells et al., 2009) to hypothesis-free machine-learning approaches^2^ (e.g., Güllich et al., 2019). Some researchers describe the talent selection approaches to be analytical and economically rational (Slack and Parent, 2006), while others have challenged the assumption that talent selection can be a rational or objective process (Cushion and Jones, 2006; Christensen, 2009; Lund and Söderström, 2017), describing the process as impulsive, irrational, atheoretical, and costly (Bar-Eli et al., 2011).

It has been acknowledged by coaches that selection and de-selection decisions are amongst the most challenging aspects of coaching (Capstick and Trudel, 2010). Not only does a wrongful selection or de-selection decision hurt the program from a performance and resource allocation perspective, but it could also lead to serious repercussions for the athlete. Pinder et al. (2013) called this wrongful inclusion or exclusion “talent wastage^3^” and proposed that a potentially large number of talented performers could be excluded from competitive sport opportunities. Once de-selected, the likelihood of an athlete reaching the elite levels of sport is greatly reduced (Huijgen et al., 2014). Moreover, athletes who have been de-selected from a team have reported feelings of anxiety, humiliation, anger, and a loss of athletic identity, sense of self, and connectedness to school (Grove et al., 2004; Barnett, 2007; Brown and Potrac, 2009; Blakelock et al., 2016; Neely et al., 2016). Over the past few years, there has been increased interest in understanding talent identification and selection with the goal of improving how these processes occur and thereby reducing talent wastage. In this paper, we summarize what is known about the factors affecting the efficacy of talent identification and selection in sport and highlight several areas where this process might be improved.

It is likely much of the “waste” is connected to the poor predictive capabilities of talent identification programs, which may be related to a number of different factors including: (a) a lack of understanding of what talent is and the way it manifests, (b) cognitive biases affecting human judgment, and (c) situational factors affecting the quality of decisions being made. With the limited research conducted directly on influences affecting talent selection in sport, this paper will explore research from other relevant domains (e.g., psychology, economics, and medicine). The present paper uses a critical review approach to extend beyond a mere description of relevant articles and to act as a “launch pad” for future development in the field, rather than answering a specific research question using a systematic approach (Grant and Booth, 2009). The aforementioned factors included for this critical review were determined through mapping review exercises to better understand the extent and gaps within the literature on the topic (Evidence for Policy and Practice Information and Co-ordinating Centre, 2006).

## Limited Understanding of Talent and How It Evolves Over Time

Arguably, one of the most fundamental issues affecting the accuracy of talent predictions is the limited understanding about the phenomenon itself. In forecasting situations, decisions are made based on the availability of information and the combined assumptions about how that information relates to future performance (Schorer et al., 2017). Although seemingly straightforward, *what* information is deemed “important” and *how* that information relates to future talent remains relatively unknown. A recent systematic review conducted on talent identification research from 1990 to 2015 highlighted only 20 articles (from an original list of 1,696 articles) examined the differences between highly skilled and less-skilled athletes over a period of 1 year or more (Johnston et al., 2018). Results from this review speak to a lack of comparative, longitudinal studies and expose the limited knowledge we have about talent and how it can be effectively measured. Longitudinal studies reduce the likelihood of a biased sample of talented individuals leading to so called “survivor effects,” found when only examining those who stay in the system and assuming they reflect qualities needed for success. Even within this limited evidence base, there is large variation in the way talent is defined and likely an even greater degree of variation in the way it is understood and applied in practice. Baker et al. (2018) suggested future research may benefit from improved operationalizations of talent in order to better evaluate the validity of this concept (see also Baker and Wattie, 2018; Bergkamp et al., 2018).

In addition to definition-related issues, our understanding of how talent develops and evolves over time is limited. This is important because sporting organizations in many nations are increasingly tasked with the identification of talent at younger and younger ages (Williams and Reilly, 2000; Abbott and Collins, 2004; Lidor et al., 2009; Mann et al., 2017). Early identification processes have been reported to begin as early as 6 years of age (Baker and Wattie, 2018). Despite the prevalence of early selection practices, and how deeply rooted they are in athlete development programs, reliable and valid early indicators of adult performance have yet to be found (Ericsson and Charness, 1994, 1995; Ericsson, 1998; Nash and Collins, 2006; Wattie and Baker, 2017; Baker and Wattie, 2018).

This is likely related to the unsupported assumption that talent follows a predictable trajectory. Goodman (1946) called this assumption the “riddle of induction,” whereby evidence from the past leads to a rule intended to predict the future. The challenge of projecting from the past is that it creates a linear and causal model in a judge’s^4^ mind which may lead to problematic and restrictive ways of thinking (Taleb, 2007). These causal relationships (between variable(s) *x* and talent outcome *y*) are difficult to justify unless meeting the criteria that variable *x* precedes *y* temporally, is reliably correlated, and has a direct correlation with *y* beyond random chance (Kennedy, 1979). In reality, the evidence for talent development is not strong enough to support causal claims. As Simonton (1999) and Howe et al. (1998) noted, being talented at a young age does not necessarily lead to being talented later in life, or vice versa. Additionally, many of the qualities that distinguish top athletic performance in adults may only emerge later in development (Bloom, 1985; French and McPherson, 1999; Simonton, 1999; Morris, 2000). Moreover, some believe talent^5^ emerges out of dynamic networks with multiple components and multiple interactions speaking to the “unpredictable” nature of talent (Simonton, 1999; Phillips et al., 2010; Den Hartigh et al., 2016). The literature focusing on the accuracy of predictions in other disciplines such as meteorology and stock trading has highlighted a relationship between time and accuracy of predictions, whereby time is inversely related to accuracy (Swets, 1988; Silver, 2012). Poincaré (1913) argued that projections into the future require increasing amounts of knowledge and precision about the process under examination, as the rate for possible error grows rapidly. Unfortunately, the degree of precision necessary for effective predictions for talent in sport does not match the current degree of knowledge. For example, Silver (2012) noted that performance statistics taken from high school or college level in baseball hold barely any predictive power for future performances in the minor (AA and AAA) and major leagues. There is an added challenge for forecasters, and specifically talent selectors, to make predictions using variables that are in a state of change (Pearson et al., 2006; Elferink-Gemser et al., 2007; Vaeyens et al., 2008). For example, a female athlete between the ages of 11–14 is thought to be at her “peak height velocity,” a time characterized by rapid changes in height and weight (Philippaerts et al., 2006). Depending on when she is assessed, it may help or hinder her chance for selection to a team as many of her attributes and capabilities may fluctuate (Vaeyens et al., 2008). Although there are a number of tests demonstrating statistically significant associations with future sport success, such tests are questionable in their ability to accurately predict talent in sport (Bahr, 2016), especially given the unstable and dynamic nature of talent posing a potential infinite number of interactions to consider (Den Hartigh et al., 2016, 2018a). The combined effects of a limited understanding about talent and how it changes over time, have implications for effective talent selections. It is also likely this limited understanding is amplified by the many cognitive limitations that arise during decision-making procedures.

## Cognitive Biases, Illusions and Perceptions Affecting Judgments About Talent

Human decision-making is beset by psychological pitfalls, something that has been more widely recognized in the last few decades. In addition to being prone to biases, humans (and therefore their decisions) have been shown to be highly influenced by emotion, fatigue, hunger, and mood (Danziger et al., 2011; Johnson and Tversky, 1983; Slovic et al., 2002; Västfjäll et al., 2014). Collectively, these studies speak to the reality of the “human effect” and demonstrate the difficulty in remaining unbiased and objective, even in the most objective professions.

Put succinctly, wherever there is a requirement for human decision-making, there is potential for error. Each decision maker will have his/her own preferences and values, but some common habits exist when forming judgments: (a) there is a tendency to rely on a relatively small number of cues (*n* = 3–5), (b) many judgments follow a linear and predictable way of thinking, and (c) there is a low degree of inter-judge agreement (Hastie and Dawes, 2001). This is not to paint all judges with the same negative brush, but rather to acknowledge that even “expert” judges adopt similar ways of thinking. Some of the linear and predictable ways of thinking include a tendency to (a) forget specifics and remember generalities, (b) to store memories differently depending on the way they were experienced, (c) to be drawn to details that confirm personal beliefs, (d) to find stories and patterns in sparse data, (e) to fill in characteristics to fit stereotypes and prior histories, and (f) to project current mindsets into the past and future (Benson, 2016). In the decision-making process, from formulating judgment to executing a decision, there are many opportunities for cognitive shortcuts. Some of these shortcuts (i.e., heuristics) can be helpful in the decision-making process and some can be hurtful (Simon et al., 2017). To highlight how these biases likely affect judgments regarding talent, we describe (a) personal preferences and intuition, (b) framing and the endowment effect, (c) the illusion of confidence, and (d) the primacy effect.

### Personal Preferences, Beliefs, and Intuition

Perhaps the greatest influences affecting the selection of athletes are the preferences, beliefs and/or goals of the talent selector (Christensen, 2009; Jokuschies et al., 2017). A talent selector’s lived experiences along with the education and environment he/she was exposed to (known as tacit knowledge), are likely to influence the types of athletes selected (Cushion and Jones, 2006; Christensen, 2009; Lund and Söderström, 2017). However, few researchers have attempted to study how talent selectors develop, access, and utilize, knowledge at appropriate times and how that knowledge plays a role in their decision-making (for exceptions, see Cushion and Jones, 2006; Vrljic and Mallett, 2008; Christensen, 2009; Mills et al., 2012; Lund and Söderström, 2017). Simon (1955) observed that decision makers identify a relatively small number of cues to form simplified models to evaluate complex problems. This model is believed to reflect the decision-makers’ personal preferences, beliefs, or goals^6^ (Lund and Söderström, 2017). In a sport-related example, Bucci et al. (2012) recognized that coaches selected their “best” athletes based on their similarity to the coaching staff, and the alignment to the staff’s ideologies/values. This type of approach speaks to the importance of personal values and their role in influencing decisions.

Under conditions of uncertainty, talent selectors often have incomplete information (e.g., they may not know a player very well, may not know very much about their past performances, and may be uncertain about how the athlete will perform at a higher level). As a result, decisions may be influenced by a decision-maker’s intuition (Nash and Collins, 2006; Plessner et al., 2011). These automatic-thinking processes can be time-efficient strategies but can also lead to systematically flawed decision-making outcomes. Nash and Collins (2006) argued that as expertise grows, the decision-making process becomes less well-defined in a talent selector’s mind. Similarly, Davids and Myers (1990) believe that with increased expertise, there is a greater reliance on intuitive feelings. It is also important to consider talent selectors may think they are using intuition to make a decision, but in reality, have a well-defined approach for selection, but difficulty articulating their thoughts (Nash and Collins, 2006).

In a study exploring the sources of information coaches use to assess talent, Christensen (2009) found coaches tend to use their visual experience to recognize patterns to help identify talent, referred to as the “coaches’ eye”. What is not well known is whether this coaches’ eye differs from intuition and whether it is helpful in increasing the accuracy of talent predictions. It is possible the coaches’ eye is a superior selection approach as expert coaches have extensive domain specific knowledge (Côté et al., 1995; Nash and Collins, 2006), and are believed to think in different ways than non-experts (Chase and Simon, 1973; North et al., 2011). For example, it has been recognized that skilled and less-skilled individuals search for information and perceive their environment in a very different manner (Raab and Johnson, 2007^7^; McRobert et al., 2009). Ericsson and Kintsch (1995) proposed skilled individuals have complex task-specific encoding skills and memory retrieval structures that differ from less-skilled individuals. However, further research is required to better understand what the “coaches’ eye” entails and its relative strengths and weaknesses in talent selection decisions (Williams and Reilly, 2000; Andersson et al., 2005; Vaeyens et al., 2008; Jokuschies et al., 2017).

### Framing and the Endowment Effect

Many professional sports use a “draft” to select newly eligible athletes to their team. Because of the considerable cost of athlete salaries in many professional sports, draft selections come with considerable economic risk, as demonstrated by the discrepancy in salaries across draft rounds. It has been reported that the first overall pick can sign an initial contract of up to four times the amount of the last pick even in the same (i.e., first) round (Massey and Thaler, 2010). In their classic studies on the psychology of decision-making, Tversky and Kahneman (1981) demonstrated that the outcome of a decision depends on how the scenario is framed. For example, a question framed in terms of losses, often leads to a person making “risk adverse” decisions. In contrast, a question posed in terms of gains, often leads to more “risk seeking” decisions (Tversky and Kahneman, 1981). Given the generality of this cognitive bias, it is possible a talent selector who is told to acquire an athlete (framed in terms of gains) may become “risk seeking,” which could lead to overvaluing the desired player. On the other hand, if the talent selector is asked to trade an athlete on the team (framed in terms of losses), they may become more “risk adverse.” This relates to a recognized phenomenon called the “endowment effect,” where there is a tendency to overvalue things already owned and to undervalue things that are not owned (Kahneman et al., 1990, 1991).

Both “risk aversion” and the “endowment effect” are closely related to the “sunk-cost bias”, whereby the investment in something (time, energy, or money) leads to the feeling that one must get a worthy return on his/her investment (e.g., the feeling of obligation to drive to the symphony in a horrible snow storm only because a ticket had already been purchased). Often a sunk cost is accepted in an effort to avoid social or personal disproval. In sport, a talent selector may turn down a trade that he/she might have otherwise made due to the influence of the “endowment effect” or the “sunk cost bias,” which subsequently may affect the accuracy of talent selections (for examples, see Staw and Ross, 1989; Staw and Hoang, 1995; Lewis, 2016). Similarly, substantial monetary investments (mostly through signing bonuses) have been shown to lead NFL coaches to provide more playing opportunities to players drafted in higher rounds, despite these players do not outperform their counterparts selected in later rounds of the draft (Keefer, 2017).

### The Illusion of Confidence

A relationship has been observed between perceived level of confidence and the accuracy of predictions. In many domains, confidence exceeds accuracy (Lichtenstein et al., 1982; Keren, 1991; McClelland and Bolger, 1994); examples include physicians’ predictions of pneumonia (Christensen-Szalanski and Bushyhead, 1981), economist’s quarterly forecasts of recession (Braun and Yaniv, 1992), and chess players’ predictions of their opponents’ moves (Griffin and Tversky, 1992). This overconfidence is believed to lead to relatively systematic errors in predictions (Kahneman and Tversky, 1973; Alpert and Raiffa, 1982) as it has been suggested those with increased levels of confidence are prone to greater levels of dispositional biases and/or illusions to avoid social disproval (Tsay and Banaji, 2011). For instance, the “confirmation bias” (Nickerson, 1998) is common in overly confident judges where there is a tendency to search for, focus on, and remember information in a way that corroborates his/her hypothesis. In another example, overly confident forecasters fell victim to “retrospective distortion” more frequency than their less-confident counterparts. Known as the “hindsight bias,” or the “knew it all along” effect, retrospective distortion is characterized by the tendency to see past events as being more predictable than they really are (Fischhoff and Beyth, 1975; Hertwig et al., 2003).

Psychologists Robyn Dawes, Paul Meehl, and Phil Tetlock are known as the “expert-busting” researchers (Lewis, 2016). Their studies have exposed an “expert problem” whereby, those who have a “bigger” reputation are often worse predictors than those who hold a less notable reputation in certain fields (Camerer and Johnson, 1997; Tetlock, 2005, 2016; Taleb, 2007). In his book, Meehl’s (1954) reviewed 20 studies showing that well-informed experts who predicted outcomes were not as accurate as a simple algorithm that added up objective data. A study by Tetlock (2005) surveyed political pundits who were asked to make predictions for multiple major events in 1980s and 1990s. Findings revealed the experts were only slightly more successful than random chance, worse than a basic statistical model of prediction, but reported high levels of overconfidence. Schorer et al. (2017) compared predictions between regional and national coaches in predicting the future performance of handball athletes and found there was little difference between levels of coaches. Last, newspaper tipsters were not found to be more successful in predicting soccer matches than the simple strategy of assuming home wins (Forrest and Simmons, 2000). It is important to acknowledge that not all of these examples directly relate to coaching expertise and their decision-making accuracy; however, it does raise important questions about the “expert effect” and how it might influence the accuracy of predictions for talent. These examples are not intended to allude that all experts are poor decisions makers (for counter examples see Tetlock, 2016), rather to highlight the importance of exploring confidence as a potential factor or proxy for illogical or error-filled decision-making processes for talent selection in sport.

### Time to Make a Prediction and the Primacy Effect

In most cases, talent selectors have limited time to gather information about an athlete and whether he/she should be accepted to the team. It can be common for a talent selector to only have two or three interactions with an athlete before a judgment and subsequent decision is made. In junior ice hockey (e.g., House League), coaches draft players based on try outs over the span of a few days (Tromp et al., 2013). In Netherlands, talent identification and development programs for soccer at the youth and adolescent levels begin after the first day of training and subsequent de-selections are made on a daily basis thereafter (Huijgen et al., 2014). With such a constrained amount of time, a coach’s ability to make informed assumptions about an athlete’s potential is compromised. This is especially true if that athlete is not performing at his/her “best” during the assessment period (i.e., injury, personal circumstances, etc.).

Additionally, Nickerson (1998) noticed that a decision maker’s thoughts are often dominated by his/her initial impressions, known as the “primacy effect.” This primacy effect may hold particular interest to decision makers because a talent selector’s first impression may be the only impression that he/she remembers from a try-out or talent identification camp. If an athlete underperforms (compared to his/her standard) then that athlete may need to work even harder to impress the talent selector and to overcome the primacy effect (Silver, 2012).

## Situational Factors

In addition to the previously mentioned influences affecting talent selection, there are situational factors that affect a talent selector’s accuracy. These factors include, (a) the use of standardized testing batteries, (b) the incorporation of machine based approaches, (c) politics or policy-related issues, (d) the number and personality of people in the decision-making process, and (e) the limited opportunities for feedback to update decisions.

### Standardized Testing Batteries

To date, much of the research on talent identification has focused on the types of testing batteries used in talent identification programs (Lidor et al., 2005; Breitbach et al., 2014). Despite the focus on testing, there is little agreement on which tests reliably predict talent; moreover, very little is known about how test results influence the decision-making process. The type of testing battery, the execution and measurement of the tests, and the way the results are used may affect the accuracy of talent selection. Some of the most commonly used methods include physical and anthropometric testing (Gil et al., 2014), technical skill measurements (Williams and Reilly, 2000; Vaeyens et al., 2006; Waldron and Worsfold, 2010; Höner et al., 2017), assessment of tactical (Kannekens et al., 2011) and perceptual cognitive capabilities (Ward and Williams, 2003; Roca et al., 2012; Causer and Ford, 2014), as well as evaluation of psychological factors (Toering et al., 2009). In most studies, measurements have been “unidimensional” in nature with a focus on one area of performance (e.g., solely the physiology of the attribute). Within those unidimensional studies, there is little agreement on whether those factors reliably predict successful performance (Lidor and Lavyan, 2002; Lidor et al., 2005; Johnston et al., 2018). Moreover, the appropriate weight to give to an athlete’s scores on different tests is largely unknown. For example, if an athlete tests poorly on an agility drill, but outperforms her teammates in a scrimmage, how do these scores affect the coach’s evaluation of that athlete relative to selection? In essence, these issues relate to coaches’ “sensitivity” and “specificity” when classifying athletes (Parikh et al., 2008). If a coach has a high level of sensitivity, he/she has an increased likelihood of correctly selecting athletes who meet or exceed expectations. Similarly, a coach who has a high degree of specificity has an increased accuracy of de-selecting athletes who would have been true under-performers. Although, the ultimate level of sensitivity and specificity is difficult to determine because there is little way of knowing if the “correct” decision has been made (i.e., it is nearly impossible to determine whether the right athletes were selected or de-selected). This speaks to the importance of a coach knowing his/her comfort level with making a type 1 or type 2 error in the process. Until tests for identification and selection are sensitive enough to reflect the physical, psychological and cognitive aspects of sport, in both elite and lower levels of competition, caution should be taken to avoid an over-reliance on testing measures to categorize individuals as “talented” or “untalented.”

Pinder et al. (2013) proposed that a driving factor affecting the low degree of reliability is related to inappropriate measurements of talent. Many talent identification programs are accused of adopting testing batteries that do not accurately represent the sport demands (Pinder et al., 2013). This is often combined with reliance on a relatively small number of heavily weighted variables measured in isolation from the sport context (Abbott et al., 2005). It is also likely there is variability in the extent to which the same component contributes to successful performance across different sport domains, levels of competition, age of athletes, or even different playing positions within the same sport (Bergkamp et al., 2018). These non-representative, highly variable, and reductionist approaches have been recognized for limiting the ability to accurately test and identify talented athletes (for recent reviews on the fidelity of testing batteries see Bergkamp et al., 2019 and Den Hartigh et al., 2018b). A call from researchers has asked for more ecologically valid and representative designs that mirror the position-specific demands of the sport to adequately assess athletic performance (Pinder et al., 2013; Den Hartigh et al., 2018b). By rigorously studying an athlete’s development over a substantial period of time (more than one season) through a multidimensional lens (physiology, perceptual cognitive ability, psychology, and motor task ability, etc.), there is a greater likelihood for understanding the capabilities and limitations of measuring talent.

### Machine-Based Approaches

One of the ways researchers and practitioners have tried to minimize the degree of variability due to human error and bias is by incorporating computer-based modeling. This can be done in two ways. First, many talent selectors at the professional level are turning to a blended approach to athlete selection, combining human judgment with “artificial intelligence.” In many professional sport leagues, the current debate is not whether statistics should be used in the decision-making process, but rather which statistics are best (Lewis, 2003, 2016). However, while this technique is starting to trickle down to lower levels of sport participation, little is known to date about the efficacy of prediction modeling for selection at younger ages of sport performance.

A second approach uses the computational power of modern technology to recognize more complex patterns of variable interaction. For instance, Güllich et al. (2019) used a machine-learning approach to identify patterns in key factors that distinguished super-elite from elite athletes in the United Kingdom. Conceptually, this approach considers possible patterns and interactions amongst a vastly superior number of variables than can be considered in traditional analyses. This approach among others (e.g., Maymin, 2017) allows researchers to test more complex and dynamic models without the statistical power requirements of approaches such as Analysis of Variance or multiple regression. What is yet to be determined is whether collecting and analyzing a greater number of variables can in fact lead to better predictions for sporting talent.

With growing reliance on technology, more research is illuminating the relative advantages and disadvantages of using computers to help in forecasting situations. For instance, more rapid and reliable decisions are not necessarily better decisions. Poor initial input will compromise the accuracy of predictions (i.e., the “garbage in, garbage out” analogy). Additionally, computers are reliant on sound and accurate models to form the basis for the coding underpinning the analysis and many (e.g., Abbott et al., 2005; Baker et al., 2018) have argued that current models of sporting talent are too simplistic. Interestingly, however, with the appropriate information, simple computer models have been shown to be very good at making predictions (Bejnordi et al., 2017). Even when people claim their mental models are more complex than a simple linear equation, an overwhelming amount of empirical research suggests that a basic equation does a surprisingly good job of capturing their judgment habits and in most cases, outperforms their predictions (Meehl, 1954; Sawyer, 1966; Goldberg, 1968; Einhorn, 1972; Libby, 1976; Cooksey, 1996; Grove and Meehl, 1996; Grove et al., 2000; Den Hartigh et al., 2018b). The studies also illuminated that experts correctly selected the variables that were important in making predictions, but surprisingly, the linear model that combined the variables and their associated weights outperformed the global judgments of the same experts. It will be important to learn how computer systems can help evaluators make talent selection decisions. More specifically, it will be important to learn how they may help to overcome the cognitive constraints explored in the section below.

### Political and Policy-Related Issues

The accuracy of talent selections may also be related to the politics at play. For a talented athlete, there may be many reasons he/she is not selected to the team. For example, some teams must include a certain number of domestic and international players and may be forced to make decisions to reach certain quotas (Aarons, 2018). In another example, a coach or other staff member with a son/daughter in the sport may directly or indirectly influence the selection of his/her son/daughter at the expense of a more “suitable” athlete to the team.

There is also a natural tendency to listen to others who are in positions of power, who exude confidence, and have overbearing personalities (Surowiecki, 2004). For instance, people trust more “confident” financial advisers over those who are less “confident” even when their track records were identical (Tetlock, 2016). It is possible that a talent selector will follow the advice or encouragement of a colleague or a parent because of the perception of “authority” or perceived confidence. Knight and Harwood (2009) noted that youth sport coaches were concerned about parents’ reactions, and reported parents being a stressor in the selection process. It appears that coaches strive to make fair decisions about their selections, which could lead to a decision being made based on the desire to appease others (i.e., parents, staff, and friends).

### Number of People Involved in Decision-Making

The accuracy of predictions is thought to be influenced by the number of people involved in the decision-making process. There is strong empirical and theoretical evidence demonstrating a benefit from aggregating different forecasts (Surowiecki, 2004; Silver, 2012; Budescu and Chen, 2014; Martire et al., 2018). Across a number of different disciplines, from medicine to political polling, the averaging of forecasts (rather than relying solely on one forecast) has been found to reduce error (Surowiecki, 2004; Yaniv, 2004; Hastie and Kameda, 2005; Silver, 2012). The exact number of forecasters needed to improve the accuracy of a prediction is still debated, but it appears there may be a “goldilocks-zone” between having two few and too many forecasters. Multiple advantages have been highlighted from applying the principles of “the wisdom of the crowd” and aggregating forecasts such as (a) maximizing the amount of information available to craft a judgment, (b) reducing the potential impact of an extreme source of information that may be unreliable (Ariely et al., 2000; Johnson et al., 2001), and (c) increasing the credibility and validity of the aggregation process (Wallsten and Diederich, 2001).

If individuals who evaluate sporting talent behave similarly to other prediction domains, selectors who include additional personnel from different perspectives in the decision-making process, may positively influence the accuracy of talent selections. This is likely dependent upon resources, program structure and situation constraints. For example, some programs may only have one coach (sometimes a parent) who is tasked with selection decisions, whereas other programs (e.g., Netherlands) have been reported to include trainers, coaches and technical staff in the selection process for adolescent soccer players (Huijgen et al., 2014). With an increased number of judges in the selection process, there is a greater likelihood of making a more rational and less biased prediction (Surowiecki, 2004). This statistical phenomenon known as “the wisdom of crowds” is rooted in the mathematical aggregation of individual estimates (Lorenz et al., 2011; Surowiecki, 2004. Under the right circumstances the wisdom of crowds effect can lead to surprisingly close estimations and predictions in different domains such as stock markets, political elections, and quiz shows (Surowiecki, 2004). Caution should be taken, however, as more people in the decision-making process does not always result in better decisions. For example, it has been demonstrated that even mild social influences can negatively influence the wisdom of crowds effect in simple estimation tasks (Lorenz et al., 2011).

### Feedback Opportunities

The nature of talent identification programs limits the ability for a talent selector to observe his/her accuracy in making predictions. For a prediction to be considered “correct,” a mechanism for feedback must be available to the decision maker. Many coaches (especially at lower levels of competition) may only have one season with an athlete and therefore have limited knowledge of whether that athlete continued in competitive sport. It is possible that opportunities to receive feedback in such a long developmental pathway could be a limiting factor affecting accuracy rates. For instance, Tetlock (2016) noted police officers were not nearly as good as they thought they were at identifying guilty subjects from innocent ones, despite the fact they spend substantial amounts of time on such tasks as part of their duties. This is thought to relate to the fact that it often takes months or years for charges to be laid, trials to be run, and verdicts to be made. Even when there is a resolution, many factors may have influenced the outcome, and during that process, officers seldom receive clear feedback about whether their judgment was accurate (Tetlock, 2016). Conversely, a meteorologist is provided fairly instant feedback and their accuracy rates continue to improve. Future predictions could benefit from further research examining the possibilities for talent selector feedback.

## Can Better Forecasts Be Made?

It may be true that talent in sport cannot be studied with the rigor of chemistry or geology, but that should not necessarily mean that a reliance on intuition and a “coach’s eye” should be encouraged. Drawing inferences from other disciplines will only lead us so far, which is why it will be important for future research to study decision-making in the specific and varied contexts of sport. Part of the solution could be to place a greater emphasis on studying the process of decision-making for talent selection rather than the outcome of the decision itself (i.e., what are the sources of information talent selectors use when shaping their beliefs about players’ skill levels?). This includes encouraging talent selectors to explicitly state their rules for decision-making and to add a weight to the judgment inputs used in their mental modeling (Musculus and Lobinger, 2018). Additionally. evidence from decision-making research encourages judges to express and quantify uncertainty in predictions by reporting a margin of error (Hastie and Dawes, 2001). This approach encourages judges to gather evidence in a meaningful way and provides a method to calibrate outcomes for feedback purposes (e.g., out of all the times you said there was a 40% chance, how often did that actually occur?). Combined with the recognition that our assumptions, biases and illusions play a role in distorting and interpreting signals we receive (Taleb, 2007; Silver, 2012), this approach to talent selection may help selectors better understand their own processes, give context and meaning to their approaches, and provide a method for checking accuracy.

As we enter the age of Big Data, with information and processing power increasing at startling rates, it is important to consider how we can incorporate computer-based modeling in a responsible way. It will be important to find a balance between combining the best of “artificial intelligence” and human capabilities to create models that are detailed enough to be helpful, but to also accurately represent the phenomenon (Silver, 2012; Den Hartigh et al., 2018b). As noted earlier, what is still unclear is whether the collection of more variables will in fact lead to better prediction. This highlights the importance of recognizing that if you cannot make a good prediction, sometimes it can be harmful to pretend you can, especially when this involves providing feedback about potential to young athletes and/or removing them from the athlete development system.

## Conclusion

To date, the available evidence of the accuracy of talent decisions by talent selectors is not compelling (Schorer et al., 2017). In this review, we have summarized a range of potential factors that may explain, at least partially, these low accuracy rates. However, it is important to note much of this research has been done outside of sport and future work with evaluators in sport settings would help to inform our understanding of how judgments are formed and decisions are made, as well as the influences affecting them, which, in turn, has the potential to improve future predictions. With more effective decision-making procedures, it is possible to minimize talent wastage and minimize the risk of wrongfully de-selecting an athlete from the sport participation pathway.

## Author Contributions

KJ and JB substantially contributed to the review. Both authors drafted the manuscript and revised it critically, gave their final approval of the current manuscript version to be published, and agreed to be accountable for all aspects of the work in ensuring that questions related to the accuracy or integrity of any part of the work are appropriately investigated and resolved.

## Conflict of Interest

The authors declare that the research was conducted in the absence of any commercial or financial relationships that could be construed as a potential conflict of interest.

## Footnotes

1 The term “talent selector” will be used throughout the paper to capture all three classifications.

2 In 2003 the Boston Celtics of the National Basketball Association selected Brandon Hunter  based  on  the output of computer algorithm. He became the first player to be picked by an “equation” (Lewis, 2016).

3 The term “talent wastage” refers to wastage of “potential” throughout the athlete development system leading to system inefficiencies.

4 For the purposes of this paper, the term “judge” will be used to represent a person crafting a judgment for decision to be made.

5 Some studies use synonyms such as “excellence” in reference to “talent.”

6 Simon’s “administrative” model of decision-making was applied to the economics domain but may hold relevance for understanding how coaches make decisions during selection processes (Neely et al., 2016).

7 The examples provided include participants who are in a temporarily constrained visual task, which challenges the transferability of findings to other domains.
